# Serum chromogranin A correlated with albuminuria in diabetic patients and is associated with early diabetic nephropathy

**DOI:** 10.1186/s12882-022-02667-0

**Published:** 2022-01-21

**Authors:** Hui Yu, Hongping Wang, Xue Su, Aili Cao, Xingmei Yao, Yunman Wang, Bingbing Zhu, Hao Wang, Ji Fang

**Affiliations:** 1 Department of Nephrology, Laboratory of Renal Disease, Putuo Hospital, Shanghai University of Traditional Chinese Medicine, Shanghai, 200062 China; 2 Department of Endocrinology, Putuo Hospital, Shanghai University of Traditional Chinese Medicine, Shanghai, 200062 China

**Keywords:** Chromogranin A, Diabetic nephropathy, Biomarker, Microalbumin

## Abstract

**Background:**

The kidney is the main site for the removal of chromogranin A (CgA). Previous studies have found that patients with renal impairment displayed elevated concentrations of CgA in plasma and that CgA concentrations reflect a deterioration of renal function. In this study, we aimed to estimate serum CgA levels and to evaluate the role of serum CgA in the early diagnosis of diabetic nephropathy (DN).

**Methods:**

A total of 219 patients with type 2 diabetes mellitus (T2DM) were included in this cross-sectional study. These patients were classified into normoalbuminuria (*n* = 121), microalbuminuria (*n* = 73), or macroalbuminuria (*n* = 25) groups based on their urine albumin to creatinine ratios (UACRs). The degree of DN is reflected by UACR. A control group consisted of 45 healthy subjects. The serum CgA levels were measured by ELISA, and other key parameters were assayed.

**Results:**

Serum CgA levels were higher in patients with T2DM than in control subjects, and a statistically significant difference among the studied subgroups regarding CgA was found (*P* < 0.05). The levels of serum CgA increased gradually with the degree of DN (*P* < 0.001). Serum CgA levels showed a moderate-intensity positive correlation with UACRs (*P* < 0.001). A cutoff level of 3.46 ng/ml CgA showed 69.86% sensitivity and 66.12% specificity to detect DN in the early stage.

**Conclusion:**

The levels of serum CgA increased gradually with the degree of DN and can be used as a biomarker in the early detection of DN.

## Background

Diabetes is one of the fastest growing health challenges of the twenty-first century. According to recent estimates from the International Diabetes Federation (IDF), 463 million adults are currently living with diabetes. The IDF estimates that there will be 578 million adults with diabetes by 2030 and 700 million by 2045. Globally, 11.3% of deaths are due to diabetes [1]. Diabetic nephropathy (DN) is a common and serious complication of diabetes and has been shown to be a major cause of end-stage renal disease (ESRD), requiring costly renal replacement therapy in the form of dialysis or transplantation [2]. It is appreciated that up to 40% of patients with type 1 and type 2 diabetes mellitus (DM) present DN [3, 4]. Early detection and appropriate treatment are essential to prevent disability and death.

At present, the clinical diagnosis of DN relies mainly on the detection of urine microalbumin. Although urine microalbumin is the gold standard for the early detection of DN, its predictive power is still limited. In some cases, patients with type 2 diabetes mellitus (T2DM) have progressive loss of renal function before the onset of microalbuminuria. Thus, in patients with T2DM, microalbuminuria is not specific or sensitive enough for the early detection of DN [5, 6]. In recent years, important achievements have been made in finding associated biomarkers in various aspects of DN. Circulating TNF-α receptor (TNFR) levels, particularly TNFR1, are excellent predictors of ESRD in both Caucasians and American Pima Indians patients with T2DM with and without proteinuria. A large real-life epidemiological study confirmed serum uric acid (UA) may be a biomarker in DN patients with the non-albuminuric phenotype. In patients with T2DM and normal renal function, copeptin also predicted an early eGFR decline leading to chronic kidney disease (CKD)-3. Markers of tubular injury, such as Kidney Injury Molecule-1 (KIM-1), Neutrophil Gelatinase-Associated Lipocalin (NGAL), Liver-type Fatty Acid Binding Protein (L-FABP), Monocyte Chemoattractant Protein-1 (MCP-1), and Epidermal Growth Factor (EGF), have been extensively investigated as prognostic biomarkers in DN. Kim et al. found that serum extracellular vesicle (EV)-miRNA profile differs in T2DM patients with normoalbuminuria and micro/macroalbuminuria and miR-4449 was highly upregulated in albuminuric patients. Early studies in T2DM patients suggested that CKD-273 could predict both development and progression of albuminuria. However, they still cannot replace proteinuria [7]. The identification of new biomarkers that can be used as alternatives to or together with routine biomarkers in the early detection of DN is still urgently warranted. In addition, new DN biomarkers may also provide new insight into the pathophysiological mechanisms leading to complications, which are still not fully understood.

Chromogranin A (CgA) is the main member of the chromogranin family and is an acidic glycoprotein consisting of 439 amino acids with an approximate molecular mass of 48 kDa. CgA is used as a diagnostic marker of neuroendocrine tumors and to monitor tumor progression or regression during treatment [8]. CgA also has a role as a biomarker in neurodegenerative and neuropsychiatric diseases, hypertension, cardiovascular disease, heart failure and renal and liver failure [8, 9]. The kidney is the main site for the removal of CgA, and it is retained in serum with declining renal function [10, 11]. In patients with renal failure, serum CgA increases much more than creatinine and the other studied low-MW proteins [12]. CgA was shown to be increased in diabetic patients [13, 14], but its relationship with DN has not been clarified. In this study, we aimed to estimate serum CgA levels and to determine the sensitivity and specificity of this biomarker for the early detection of DN.

## Methods

### Patients and samples

**Fig. 1:**
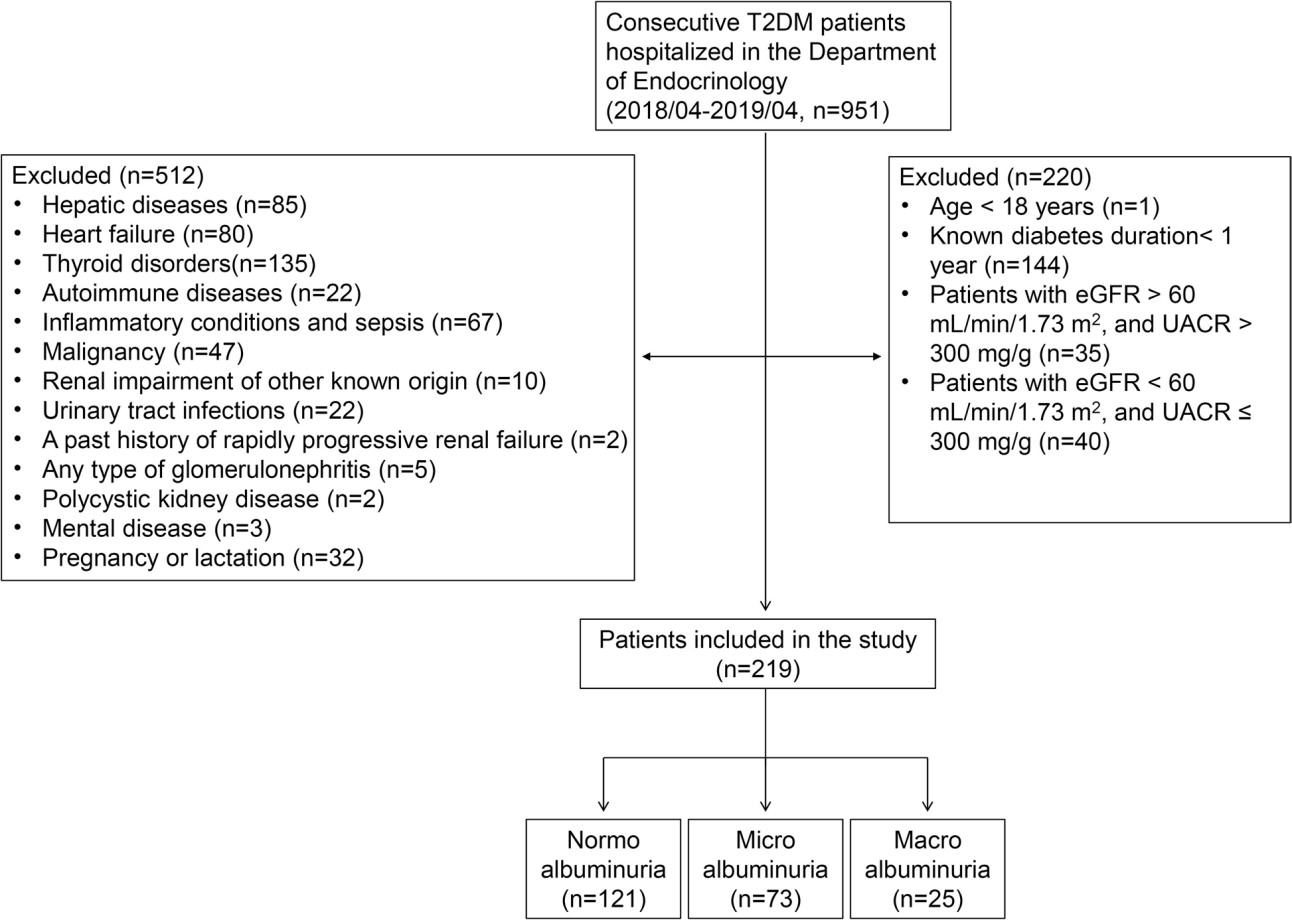
Flow chart of patient enrollment. (T2DM, type 2 diabetes mellitus; UACR, urine albumin to creatinine ratios)

In this cross-sectional study, 219 patients with T2DM (per the 2007 American Diabetes Association diagnostic criteria) who were hospitalized in the Department of Endocrinology, Putuo Hospital, Shanghai University of Traditional Chinese Medicine, China, between April 2018 and April 2019 were selected. This study was approved by the Ethics Committee of our hospital (approval number PTEC-A-2018-11-1). All participants signed informed consent. The specific inclusion criteria were as follows: (1) age ≥ 18 years; (2) known diabetes duration ≥1 year; and (3) normoalbuminuria, microalbuminuria patients (eGFR > 60 mL/min/1.73 m^2^ and UACR ≤300 mg/g) and macroalbuminuria patients (eGFR < 60 mL/min/1.73 m^2^ and UACR > 300 mg/g). Patients with hepatic diseases, heart failure, thyroid disorders, autoimmune diseases, inflammatory conditions, sepsis, malignancy, renal impairment of other known origin than DN, urinary tract infections, a past history of rapidly progressive renal failure, any type of glomerulonephritis, polycystic kidney disease, any mental disease, pregnancy or lactation were excluded from the study (Fig. 1). In the same period, 45 healthy people who underwent physical examination in our hospital were selected as controls. All groups were matched for age and sex.

All patients with T2DM were divided into three subgroups according to the urinary microalbumin to creatinine ratio (UACR): 1. patients with normoalbuminuria – UACR< 30 mg/g (*n* = 121), 2. patients with microalbuminuria – UACR 30–300 mg/g (*n* = 73), and 3. patients with macroalbuminuria – UACR> 300 mg/g (*n* = 25). Classification was performed according to the 2012 KDIGO (Kidney Disease: Improving Global Outcomes) guidelines [15]. Increased albuminuria (UACR ≥30 mg/g) was considered objective evidence of kidney disease. UACR 30–300 mg/g and eGFR > 60 mL/min/1.73 m^2^ were considered early DN; UACR> 300 mg/g and eGFR < 60 mL/min/1.73 m^2^ were considered advanced DN. The degree of DN is reflected by UACR.

Spot urine samples were collected at the first morning void. Blood samples were collected before eating in the morning, centrifuged (3000 rpm, 4 °C, 10 min) to acquire serum and stored at − 80 °C until use. Samples for measurements of serum creatinine (sCr), serum uric acid (sUA), blood urea nitrogen (BUN), fasting blood glucose (FBG), hemoglobin A1c (HbAlc), triglyceride (TG), total cholesterol (TC), low-density lipoprotein cholesterol (LDL-C), high-density lipoprotein cholesterol (HDL-C), and urinary microalbumin to creatinine ratio (UACR) levels were examined at the department of clinical examination using standard procedures. The estimated glomerular filtration rate (eGFR) was calculated using the abbreviated MDRD equation [16]. eGFR (mL/min per 1.73 m^2^) = 186 X Pcr^-1.154^ X age^-0.203^X 0.724 (if female), where Pcr is in mg/dl and age is in years. The concentration of CgA was measured using human-CgA ELISA kits (Cat# ab196271) from Abcam.

### Statistical analysis

Continuous variables with a normal distribution are reported as the mean ± standard deviation (SD), whereas skewed distributed variables are expressed as the median (interquartile range). Categorical data were summarized as proportions with frequencies. Continuous variables were compared between groups using an unpaired t test, a one-way ANOVA test, the Kruskal–Wallis test or the Jonckheere-Terpstra test where appropriate, while the chi-square test or Fisher’s exact test was used to analyze the differences in categorical variables. Logistic regression analyses were used to ascertain the predictive value of the CgA level for the presence of early DN in patients with T2DM. Considering the interaction between sCr and eGFR and their similar clinical implications, only sCr was used for multivariate logistic regression. The Spearman rank correlation test was performed to analyze the relationship between 2 quantitative parameters. Statistical analyses were conducted using SPSS software (version 23.0; SPSS, Inc., Chicago, IL, United States). Statistical significance was considered 2-tailed, with a *P* value of < 0.05.

## Results

### Clinical characteristics

All enrolled T2DM patients were categorized into three subgroups according to UACR. The demographic and laboratory features of the patients as well as the healthy subjects are reported in Table 1. Our results showed that there was a significant difference among the subgroups of patients regarding SBP, known diabetes duration, UACR, sCr, BUN and eGFR. However, there was no significant difference among the subgroups of patients regarding sex, age, DBP, FBG, HbA1c, sUA, TC, TG, HDL-C and LDL-C. Comparing the clinical and laboratory data among the subgroups of patients and the healthy subjects, there was no significant difference in sex, age, TC and LDL-C levels.

**Table 1:** Comparison of clinical and laboratory data among subgroups of patients with T2DM divided according to UACR and healthy subjects

	Heathy controls (*n* = 45)	Normo albuminuria (*n* = 121)	Micro albuminuria (*n* = 73)	Macro albuminuria (*n* = 25)	^a^*P*-value	^b^*P*-value
Male, n (%)	26(57.8%)	72(59.5%)	38(52.1%)	15(60.0%)	0.768	0.568
Age (years)	63.40 ± 10.38	62.51 ± 10.34	65.18 ± 13.52	64.48 ± 10.85	0.445	0.277
SBP (mmHg)	117.44 ± 7.04	129.50 ± 12.15^&&^	132.12 ± 14.41^&&^	141.60 ± 17.68^&&^**^#^	< 0.001	< 0.001
DBP (mmHg)	74.00 ± 6.79	79.41 ± 7.76^&&^	78.96 ± 9.47^&^	79.88 ± 6.70^&^	0.001	0.874
FBG (mmol/L)	4.81 ± 0.42	8.63 ± 4.14^&&^	8.79 ± 3.50^&&^	9.10 ± 2.76^&&^	< 0.001	0.850
HbA1c (%)	5.50 ± 0.37	9.41 ± 2.40^&&^	9.88 ± 3.19^&&^	9.45 ± 2.17^&&^	< 0.001	0.479
Known diabetes duration (years)	/	8.00(3.00, 15.00)	10.00(6.00, 20.00)**	18.00(13.00, 20.00)**^#^	/	< 0.001
UACR (mg/g)	3.59(2.04, 5.75)	8.55(5.30, 14.07)^&&^	77.69(48.49, 166.00)^&&^**	834.05(614.33, 2103.99)^&&^**^##^	< 0.001	< 0.001
sCr (umol/L)	66.73 ± 12.19	65.07 ± 13.82	72.07 ± 18.48*	137.04 ± 49.66^&&^**^##^	< 0.001	< 0.001
BUN (mmol/L)	5.11 ± 0.96	5.58 ± 1.45	7.08 ± 2.81^&&^**	8.91 ± 4.33^&&^**^#^	< 0.001	< 0.001
sUA (ummol/L)	308.84 ± 71.81	325.74 ± 94.77	345.77 ± 101.29^&^	373.84 ± 103.01^&^*	0.022	0.060
eGFR (ml/min/1.73 m^2^)	102.44 ± 21.80	106.44 ± 21.12	95.31 ± 28.75*	47.53 ± 12.50^&&^**^##^	< 0.001	< 0.001
TC (mmol/L)	4.67 ± 0.72	4.68 ± 1.59	4.38 ± 1.22	4.41 ± 1.38	0.421	0.335
TG (mmol/L)	0.94(0.75, 1.33)	1.55(1.08, 2.43)^&^	1.40(0.99, 2.12)^&^	1.66(1.13, 2.63)^&^	< 0.001	0.360
LDL-C (mmol/L)	2.97 ± 0.84	2.93 ± 1.15	2.69 ± 0.84	2.84 ± 1.00	0.373	0.319
HDL-C (mmol/L)	1.41 ± 0.28	1.13 ± 0.35^&&^	1.11 ± 0.31^&&^	1.06 ± 0.27^&&^	< 0.001	0.660
CgA (ng/mL)	1.89(1.42, 2.47)	2.26(1.38, 4.12)^&^	4.83(2.63, 6.46)^&&^**	6.35(4.91, 7.54)^&&^**^#^	< 0.001	< 0.001
ACEI/ARB, n (%)	0(0.0%)	34(28.1%)^&&^	25(34.2%)^&&^	18(72.0%)^&&^**^#^	< 0.001	< 0.001

Notes

*SBP* systolic blood pressure, *DBP* diastolic blood pressure, *FBG* fasting blood glucose, *HbA1c* hemoglobin A1c, *UACR* urine albumin to creatinine ratio, *sCr* serum creatinine, *BUN* blood urea nitrogen, *sUA* serum uric acid, *eGFR* estimated glomerular filtration rate, *TC* total cholesterol, *TG* triglyceride, *LDL-C* low density lipoprotein cholesterol, *HDL-C* high density lipoprotein cholesterol, *CgA* Chromogranin A

^a^*P*-value: comparisons among the healthy controls group, the normoalbuminuria group, the microalbuminuria group and the macroalbuminuria group;

^b^*P*-value: comparisons among the normoalbuminuria group, the microalbuminuria group and the macroalbuminuria group

^&^*P* < 0.05

^&&^*P* < 0.001 vs. healthy controls

^*^P < 0.05

^**^P < 0.001 vs. normoalbuminuria group

^#^*P* < 0.05

^##^*P* < 0.001 vs. microalbuminuria group

### Comparison of serum CgA levels among subgroups of patients with T2DM and healthy subjects

**Fig. 2:**
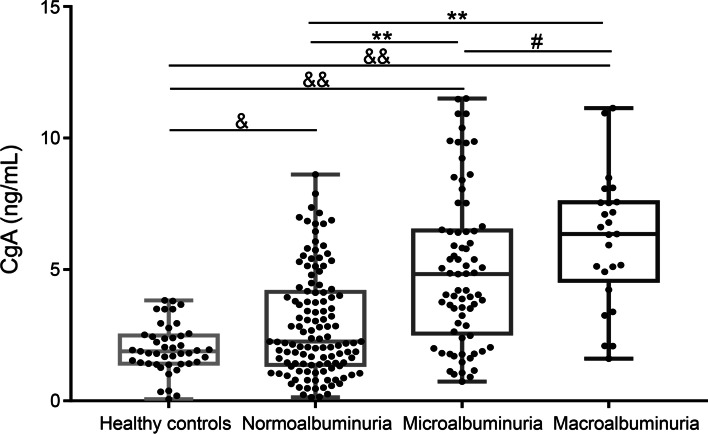
Comparison of serum CgA levels in subgroups of subjects divided according to UACR and healthy subjects. (& *P* < 0.05, && *P* < 0.001 vs. healthy controls; ** *P* < 0.001 vs. normoalbuminuria group; # *P* < 0.05 vs. microalbuminuria group)

A comparison of serum CgA levels among subgroups of patients divided according to UACR and healthy subjects was performed using the Kruskal–Wallis test. The serum CgA levels were higher in patients with T2DM [3.52 (1.79, 5.67) ng/mL] than in healthy subjects [1.89 (1.42, 2.47) ng/mL] (*P* < 0.001), and a statistically significant difference was found among the normoalbuminuria group [2.26 (1.38, 4.12) ng/mL], the microalbuminuria group [4.83 (2.63, 6.46) ng/mL] and the macroalbuminuria group [6.35 (4.91, 7.54) ng/mL] regarding serum CgA levels (*P* < 0.001). A nonparametric Jonckheere-Terpstra test showed that CgA increased gradually with the degree of DN (Z = 6.567, *P* < 0.001). The results are shown in Fig. 2 and Table 1.

### Correlation between serum CgA levels and clinical and laboratory data

The Spearman rank correlation test demonstrated that there was a moderate-intensity positive correlation between serum CgA levels and known diabetes durations and UACRs in all subjects (*P* < 0.001). Weak correlations were found between serum CgA levels and FBG, HbA1c, sCr, BUN, eGFR, and HDL-C (all *P* < 0.05), and there was no correlation between serum CgA levels and age, SBP, DBP, sUA, TC, TG, or LDL-C in all subjects. There was a moderate-intensity positive correlation between serum CgA levels and UACRs in patients with T2DM (*P* < 0.001). Weak correlations were found between serum CgA levels and known diabetes duration, DBP, HbA1c, sCr, BUN, and eGFR (all *P* < 0.05), but there was no correlation between serum CgA levels and age, SBP, FBG, sUA, TC, TG, LDL-C, or HDL-C in patients with T2DM. The correlation coefficients between serum CgA levels and clinical and laboratory data are presented in Table 2.

**Table 2:** Correlation between serum CgA and clinical and laboratory data of subjects

Clinical and laboratory data	CgA (in all subjects) (*n* = 264)		CgA (in patients with T2DM) (*n* = 219)	
	r	*P*	r	*P*
Known diabetes duration (years)	0.302	< 0.001	0.171	0.011
Age (years)	0.066	0.286	0.053	0.434
SBP (mmHg)	0.099	0.110	−0.029	0.673
DBP (mmHg)	−0.084	0.172	−0.170	0.012
FBG (mmol/L)	0.240	< 0.001	0.128	0.060
HbA1c (%)	0.285	< 0.001	0.144	0.033
UACR (mg/g)	0.441	< 0.001	0.408	< 0.001
sCr (umol/L)	0.163	0.008	0.188	0.005
BUN (mmol/L)	0.229	< 0.001	0.202	0.003
sUA (ummol/L)	0.061	0.322	0.049	0.470
eGFR (ml/min/1.73 m^2^)	−0.227	< 0.001	−0.252	< 0.001
TC (mmol/L)	−0.118	0.056	−0.109	0.107
TG (mmol/L)	0.035	0.572	−0.049	0.473
LDL-C (mmol/L)	−0.109	0.076	−0.105	0.120
HDL-C (mmol/L)	−0.124	0.044	−0.027	0.689

*SBP* systolic blood pressure, *DBP* diastolic blood pressure, *FBG* fasting blood glucose, *HbA1c* hemoglobin A1c, *UACR* urine albumin to creatinine ratio, *sCr* serum creatinine, *BUN* blood urea nitrogen, *sUA* serum uric acid, *eGFR* estimated glomerular filtration rate, *TC* total cholesterol, *TG* triglyceride, *LDL-C* low density lipoprotein cholesterol, *HDL-C* high density lipoprotein cholesterol, *CgA* Chromogranin A

### Serum CgA levels were associated with the occurrence of early DN

We performed multivariate logistic regression analyses in patients with T2DM between the normoalbuminuria group and the microalbuminuria group. As shown in Table 3, multivariable logistic regression analysis revealed that serum CgA (OR 1.403, 95% CI 1.202–1.637, *P* < 0.001) and BUN (OR 1.299, 95% CI 1.045–1.616, *P* = 0.019) were independently associated with the occurrence of early DN when adjusted for age, sex, known diabetes duration, SBP, DBP, FBG, HbAlc, TC, TG, HDL-C, LDL-C, sUA, BUN, sCr and CgA. (Enter).

**Table 3:** Multivariate logistic regression analysis of factors independently associated with early DN in patients with T2DM between the normoalbuminuria group and the microalbuminuria group

Variables	*P*-value	EXP(B)	95% CI
Male	0.252	0.603	0.254–1.434
Age	0.375	0.983	0.946–1.021
Known diabetes duration	0.164	1.039	0.985–1.096
SBP	0.468	1.013	0.978–1.049
DBP	0.538	1.017	0.963–1.074
FBG	0.564	0.971	0.877–1.074
HbA1c	0.519	1.050	0.905–1.219
TC	0.686	0.850	0.386–1.871
TG	0.637	1.052	0.851–1.302
HDL-C	0.315	2.176	0.478–9.917
LDL-C	0.927	0.958	0.385–2.384
sUA	0.816	0.999	0.994–1.004
BUN	0.019	1.299	1.045–1.616
sCr	0.097	1.029	0.995–1.064
CgA	< 0.001	1.403	1.202–1.637

adjusting for age, gender, Known diabetes duration, SBP, DBP, FBG, HbA1c, TC, TG, HDL-C, LDL-C, sUA, BUN, sCr and CgA. (Enter)

Significance level was set at *P* < 0.05

*CI* confidence interval, *SBP* systolic blood pressure, *DBP* diastolic blood pressure, *FBG* fasting blood glucose, *HbA1c* hemoglobin A1c, *sCr* serum creatinine, *BUN* blood urea nitrogen, *sUA* serum uric acid, *TC* total cholesterol, *TG* triglyceride, *LDL-C* low density lipoprotein cholesterol, *HDL-C* high density lipoprotein cholesterol, *CgA* Chromogranin A

### ROC analysis

**Fig. 3:**
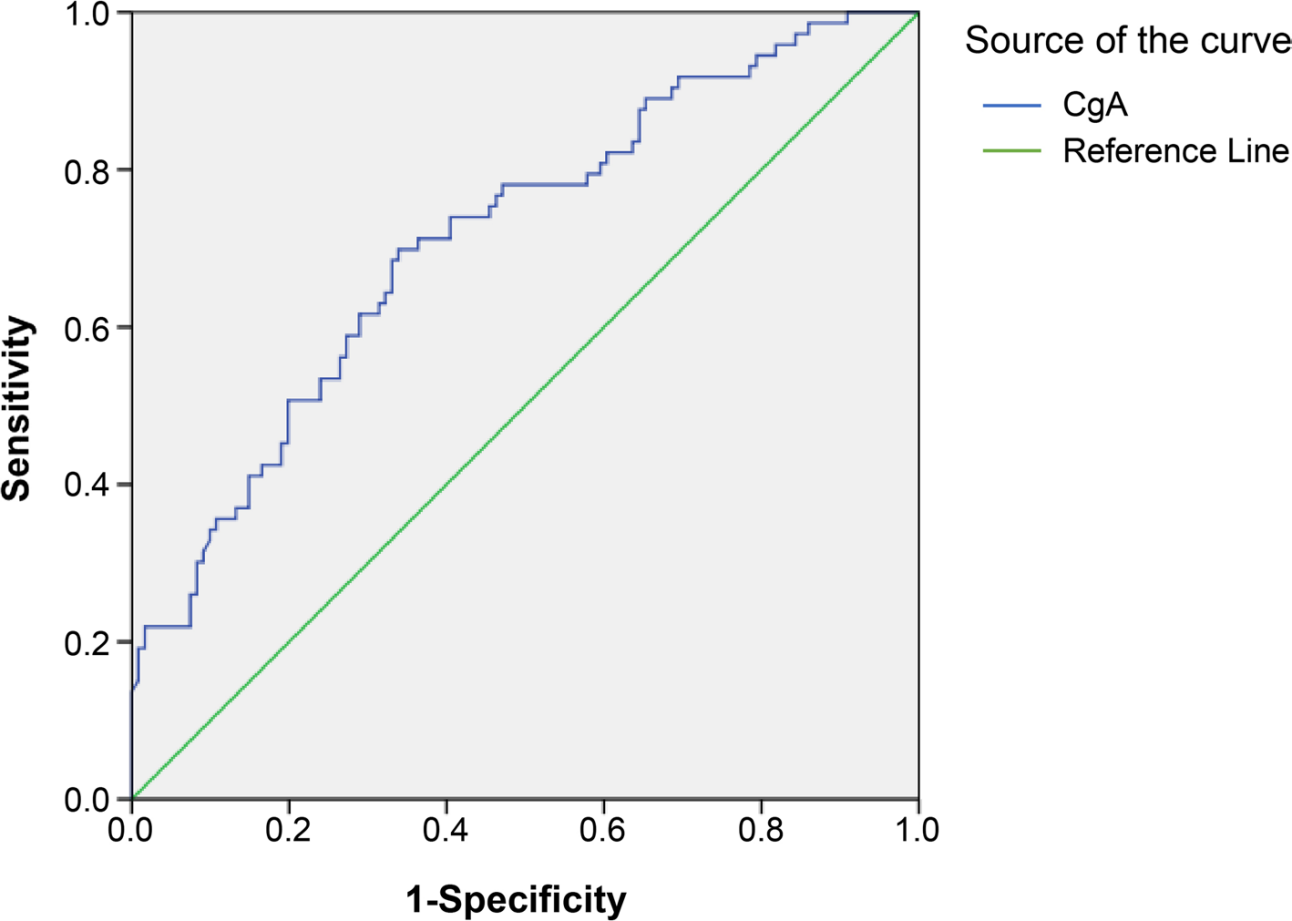
Roc curve for validity of serum CgA in prediction of early DN

ROC analysis was performed to assess the sensitivity and specificity of serum CgA as a potential biomarker in the prediction of early DN. The ROC analysis of serum CgA yielded an AUC of 0.714 (95% CI, 0.639–0.788; *P* < 0.001) in the differentiation of T2DM patients with early DN (Table 4 and Fig. 3). The diagnostic sensitivity and specificity of serum CgA for early DN were 69.86 and 66.12%, respectively, when the cutoff value was 3.46 ng/mL.

**Table 4:** Validity of serum CgA in prediction of early DN. (in patients with T2DM)

Parameters	CgA	UACR
AUC	0.714	1.000
95% CI	0.639–0.788	1.000
Youden index J	0.360	1.000
Cut-off	3.46	29.67
Sensitivity (%)	69.86	100.00
Specificity (%)	66.12	100.00
*P*-value	< 0.001	< 0.001

*CI* confidence interval, *AUC* Area Under Curve, *CgA* Chromogranin A

## Discussion

DN is an important microvascular complication of diabetes mellitus. To date, there is no satisfactory method to recognize early DN. According to KDOQI definitions [17], DN diagnosis is based on increased albuminuria and decreased eGFR. There are some shortcomings in using albuminuria as a diagnostic marker for (early) DN. The change in glomerular basement membrane structure may occur earlier than the increase in albuminuria [18]. Approximately 30–45% of T2DM patients who develop kidney disease associated with a decrease in glomerular filtration do not have increased albuminuria [19, 20]. In addition, albuminuria is not specific for DN. Hypertension or obesity may also affect the filtration barrier of the glomeruli, leading to increased albuminuria in patients with T2DM [21]. However, the treatment of hypertension usually includes renin angiotensin aldosterone inhibitors, which reduce glomerular hydrostatic pressure and cause proteinuria to be in the normal range. These factors affect the accuracy of diagnosis based on current guidelines. In DN, when the kidney is damaged, the decrease in eGFR occurs quite late. Early damage is often accompanied by hyperfiltration [22]. Therefore, both routine markers of DN (albuminuria and eGFR) reflecting glomerular damage have certain limitations.

CgA is the main member of the chromogranin family, consisting of water-soluble acidic glycoproteins. CgA was first found in secretory granules from adrenal medullary chromaffin cells and is released into the circulation after splanchnic nerve stimulation together with catecholamines [23]. CgA also exists in many endocrine and neuroendocrine cells [24], nerve cells [25] and immune cells [26]. Elevated serum CgA levels have been found in many cancers [27] and neurodegenerative diseases, such as Alzheimer’s disease [28] and Parkinson’s disease [29]. A previous study showed that CgA, similar to many other low-molecular-weight (MW) proteins, is handled by the kidney [12]. The serum levels of CgA do not change significantly until GFR is reduced to 40 mL/min. Further reduction in GFR is accompanied by a progressive increase in serum values of CgA [12]. However, serum CgA increases in renal failure more than creatinine and the other studied low-MW proteins, β2-MG (β2 microglobulin) and TATI (tumor-associated trypsin inhibitor) [12]. In animal experiments, an inverse correlation suggested a decrease in GFR with increasing plasma CgA concentration [30]. The study by Chen et al. suggests CgA-stimulation of endothelial cell exocytosis of endothelin as a possible mechanism for regulation of renal function in health and disease [31].

Our study aimed to investigate the role of serum CgA in the early diagnosis of DN in patients with T2DM. Because our groups were matched regarding age and sex, the effects of these factors on the results of serum CgA in our study were excluded. In our study, we found a significant difference among the normoalbuminuria, microalbuminuria and macroalbuminuria groups regarding known diabetes duration. This finding is consistent with other previous studies [32, 33].

In our study, the level of serum CgA in patients with T2DM was much higher than that in healthy people. A previous study documented the clinical value of measurements of CgA as a potential marker for diabetes [13]. The concentrations of plasma and salivary CgA were significantly higher in the diabetic groups than in the control group [14]. Our results are consistent with their findings.

Serum concentrations of CgA correlated strongly with eGFR [34]. The relationship between CgA and GFR is very similar to that of other substances eliminated by the kidney, such as creatinine and TATI [12]. In our results, the Spearman rank correlation test demonstrated that serum CgA was negatively correlated with eGFR not only in patients with T2DM but also in all subjects. There was a moderate-intensity positive correlation between serum CgA levels and known diabetes durations and UACRs in all subjects. So the most important finding of our study is that CgA correlated not only with eGFR and the presence of diabetes, which have already been shown in previous studies, but also with UACR and this correlation appeared even stronger than others.

We found for the first time that serum CgA increased gradually with the degree of DN and that serum CgA levels were associated with the occurrence of microalbuminuria. The multivariate logistic regression analyses in patients with T2DM between the normoalbuminuria group and the microalbuminuria group revealed that CgA levels and not eGFR were independently associated with microalbuminuria, which suggests that CgA reflects progression of renal damage even at an early stage without apparent reduction of kidney function, and thus might represent a sensitive marker of DN. One study showed that serum CgA was a poor indicator of DN since patients with T2DM and a reduced glomerular filtration rate failed to show any significant increase in serum CgA [35]. In their research, patients were divided into those without DN with normoalbuminuria (*n* = 27), patients with DN with microalbuminuria (*n* = 8), and patients with macroalbuminuria (*n* = 42). It is noteworthy that in their study, the *P* value of the difference between patients with normoalbuminuria and DN was 0.07. The failure to reach a significant difference may be due to the small sample size. Moreover, other studies have found that patients with renal impairment display elevated concentrations of CgA in plasma and that CgA concentrations reflect a deterioration of renal function [12, 36]. CgA contains multiple amino acid motifs prone to endoproteolytic cleavage, resulting in multiple processing fragments. This constitutes a biochemical challenge for accurate quantification of CgA. Several assays for measurements of CgA have been developed, but since the antibodies used detect different epitopes, the results from the assays vary considerably [37]. The assay kits we used were different from those used by OA Mojiminiyi’s team [35].

ROC analysis showed the diagnostic accuracy of serum CgA in differentiating between T2DM patients with early DN and those without DN. Using a cutoff of 3.46 ng/mL for serum CgA, we found a sensitivity of 69.86%, a specificity of 66.12%, and a diagnostic accuracy of 71.4% in predicting early DN.

The in vivo isolated cleavage products of CgA in humans include vasostatin-1 and vasostatin-2, pancreastatin (PST), WE-14, cateslytin, and catestatin (CST). All of these products have significant and specific biological effects. Recent studies suggest that WE-14, CST and PST contribute to the development of different diabetes mellitus forms [38]. At the circulation level, CST is associated with chronic heart failure, myocardial infarction, malignant arrhythmia, acute coronary syndrome and unstable angina. Vasostatin-1 and vasostatin-2 exert vasodilatory effects. Vasostatin-1 is associated with multiple myeloma, carotid artery atherosclerosis, sepsis and Takayasu’s arteritis. Vasostatin-2 increased coronary pressure in Langendorff-perfused rat hearts without affecting inotropism. Vasostatin-2 is associated with ischemic chronic heart failure and coronary artery atherosclerosis [39]. Studies on the relationship between these cleavage products of CgA and DN are rare. Further investigation should be performed to determine whether these CgA cleavage products can be used for DN detection.

The findings of this study are limited by the sample size and the cross-sectional design of the study; therefore, the direction of causality cannot be determined from these results. Moreover, the selected T2DM patients did not undergo renal biopsy. Although we formulated strict standards for excluding other patients with renal dysfunction caused by nondiabetic diseases, it cannot be ruled out that there could be patients with renal diseases other than DN selected into groups. Additional prospective studies are needed to examine associations between CgA and early DN risk.

## Conclusion

The results of this study show that serum CgA levels were higher in patients with T2DM than in healthy subjects and increased gradually with the degree of DN. CgA could also be used as a biomarker in the early detection of DN.

## Data Availability

The datasets used and/or analyzed during this study are available from the corresponding author on reasonable request.

## Abbreviations

DN: Diabetic nephropathy
T2DM: Type 2 diabetes mellitus
IDF: International Diabetes Federation
ESRD: End-stage renal disease
CKD: Chronic kidney disease
TNFR: TNF-α receptor
KIM-1: Kidney Injury Molecule-1
NGAL: Neutrophil Gelatinase-Associated Lipocalin
L-FABP: Liver-type Fatty Acid Binding Protein
MCP-1: Monocyte Chemoattractant Protein-1
EGF: Epidermal Growth Factor
EV: Extracellular vesicle
TGF: Transforming growth factor
PST: Pancreastatin
CST: Catestatin
SD: Mean ± standard deviation
TATI: Tumor associated trypsin inhibitor
MW: Molecular weight
SBP: Systolic blood pressure
DBP: Diastolic blood pressure
FBG: Fasting blood glucose
HbA1c: Hemoglobin A1c
UACR: Urine albumin to creatinine ratio
sCr: Serum creatinine
BUN: Blood urea nitrogen
sUA: Serum uric acid
eGFR: Estimated glomerular filtration rate
TC: Total cholesterol
TG: Triglyceride
LDL-C: Low density lipoprotein cholesterol
HDL-C: High density lipoprotein cholesterol
CgA: Chromogranin A
CI: Confidence interval
AUC: Area Under Curve
